# Progressive Neuropsychiatric Symptoms Following Recurrent COVID-19 Infections in a Previously Healthy Adolescent

**DOI:** 10.1155/2023/5519051

**Published:** 2023-11-08

**Authors:** Christopher Shawl, R. Hunter Clark, Matthew T. Edwards, Casey Berson, Megan Zappitelli

**Affiliations:** ^1^Prisma Health Department of Psychiatry, 701 Grove Road, Greenville, SC 29605, USA; ^2^University of South Carolina School of Medicine Greenville, 607 Grove Road, Greenville, SC 29605, USA; ^3^Mission Health Hospital, HCA Healthcare, 428 Biltmore Avenue, Asheville, NC 28801, USA

## Abstract

This is the almost 2-year-long course of a 16-year-old male without significant psychiatry history who abruptly developed symptoms of obsessive-compulsive disorder (OCD) and psychosis following a confirmed coronavirus disease 2019 (COVID-19) infection. His symptoms worsened following a confirmed reinfection with COVID-19. He responded poorly to treatment with selective serotonin reuptake inhibitors, antipsychotics, and benzodiazepines. This case highlights an emerging phenomenon of post-COVID-19 neuropsychiatric sequelae and presents a complicated diagnostic and treatment challenge. The differential for this patient was explored and outlined in detail, and the medical workup recommendations for new-onset mental status changes were reviewed as they pertain to the patient's assessment and treatment course. While there are several case reports of adolescents with abrupt-onset OCD and psychosis symptoms following COVID-19 infections, none of these reports include worsening of symptoms following reinfection, and few reports follow patients beyond initial hospitalization and treatment.

## 1. Introduction

Neurological complications associated with coronavirus disease 2019 (COVID-19) infections commonly include hyposmia, headache, dizziness, encephalopathy, delirium, and cognitive slowing [1, 2]. Neuropsychiatric complications associated with COVID-19 infections, including psychosis, depression, anxiety, and cognitive impairment, have been reported in older adults and to a limited extent in young adults and adolescents [3–7]. These complications often differ from the clinical course expected with a primary psychiatric disorder, can vary in duration of illness, have overlapping symptoms from multiple psychiatric disorders, and, therefore, do not follow well-known treatment guidelines. It has also been observed that a COVID-19 infection may also exacerbate an existing psychiatric disorder or symptoms [3–7]. We report the yearlong course of a previously healthy 16-year-old male who developed persistent symptoms of obsessive-compulsive disorder (OCD) and psychosis following a confirmed COVID-19 infection and subsequent reinfection. Similar case reports provide limited insight into ongoing treatments and responsiveness and do not include recurrence of COVID-19 infections. We hope to highlight our patient's clinical presentation in the context of recurrent COVID-19 infections and to share our experience with the inpatient and outpatient management of his complicated clinical course, as well as to delineate our thought process and approach to his treatment. Further, we will discuss our position on the necessity of shared decision-making with the patient and his family and the challenges of treatment for severe mental illness when the underlying etiology remains unclear.

## 2. Case Report

Our patient, a 16-year-old male with a remote history of rigid thinking, dyslexia, possible auditory processing challenges, and subtle difficulties with math in early childhood, presented to the children's hospital with a 10-month history of abrupt onset and progressively worsening symptoms of marked anger, aggressive behavior, obsessions, intrusive homicidal thoughts, intrusive thoughts about his sexual orientation, nonspecific auditory and visual perceptual anomalies, depersonalization, depression, hopelessness, anxiety, and sleep onset difficulty. He was initially evaluated by our child and adolescent psychiatry inpatient consultation service and then later, followed by our inpatient child psychiatry service and our outpatient child psychiatry clinic. The course of the 10-month history leading up to his first hospitalization and subsequent treatment course is outlined in Table 1 and includes five visits to his primary care pediatrician and four visits to the emergency department (ED). The patient and his family provided written and verbal consent for the publication of this case. Identifiable information has been removed from this case report.

The patient's symptoms began 3 weeks after a confirmed COVID-19 infection. He visited his pediatrician three times over the subsequent month. At the first pediatrics visit, he reported persistent shortness of breath, nasal congestion, and anxiety. His examination was normal, and no laboratory evaluation was reported. He was provided with a prednisone “burst” with 60 mg po for 5 days. He and his family were reassured, and no medical therapy was prescribed. During the second pediatrics visit, he reported worsening anxiety, hopelessness, inattention, poor sleep, and tearfulness. He was started on escitalopram 10 mg daily. At the third visit, his symptoms had progressed further to include distorted thoughts and a fear of being alone, a fear of taking showers, racing thoughts, confusion, and intrusive thoughts. Laboratory evaluation, including a complete blood count (CBC), complete metabolic panel (CMP), thyroid stimulating hormone (TSH), and C-reactive protein (CRP), were all within normal limits. He was continued on escitalopram and referred to counseling.

Five months after his first confirmed infection with COVID-19, he presented to the ED for evaluation of significant worsening of depressive thoughts, suicidal ideation, and thoughts of hurting others. He felt that the thoughts of hurting other people belonged to someone else, describing it as “someone reaching out to me.” Laboratory evaluations of CBC, CMP, ethanol, and urinalysis drug screen (UDS) were all within normal limits or negative. His evaluation did not support an imminent threat to himself or others, and he was discharged home.

Six months after his first confirmed infection with COVID-19, he returned to his pediatrician twice. He first reported additional neuropsychiatric complaints, including auditory hallucinations, derealization, cognitive slowing, and daily headaches. Laboratory evaluation of CBC, CMP, TSH, lactate dehydrogenase (LDH), uric acid, and troponin were all within normal limits. Head computed tomography (CT) did not reveal any abnormalities. He and his family were reassured and encouraged to follow up with psychiatry. He returned to his pediatrician again with complaints of difficulty with thought organization, derealization, and continued cognitive slowing and again was reassured that there was no medical cause for his symptoms.

Nine months after his first confirmed infection with COVID-19, he presented to the ED twice within 1 week for worsening symptoms and new episodes of rage, self-injurious behavior, visual and auditory hallucinations, depression and hopelessness, lack of control of thoughts, and transient suicidal ideations. Both visits to the ED included laboratory evaluation of CBC, CMP, ethanol level, and UDS, which were all within normal range or negative. At the first ED visit, he was able to contract for safety and was discharged home. At the second ED visit, he was observed for 5 days. During this time, he did not have any additional episodes of aggression and was discharged home.

Nine and a half months after his first confirmed infection with COVID-19, he returned to his pediatrician with complaints of sore throat, nasal congestion, and shortness of breath. His COVID-19 PCR test was positive, indicating that he had an active COVID-19 infection for the second time. He was instructed to quarantine and was reassured that he could follow up in the outpatient setting.

Ten months after his first infection with COVID-19 (2 weeks after his confirmed reinfection), he presented to the pediatric ED in a neighboring city. He and his parents reported that following his reinfection with COVID-19, his symptoms worsened significantly, culminating with a self-injurious episode during which he “smashed” his face on the ground. He was transferred and admitted to our pediatric inpatient hospitalist service, at which time child and adolescent psychiatry was consulted. He reported increased severity of the previously described symptoms. He reported intrusive thoughts about violence, thoughts about his sexual orientation (having thoughts that he was homosexual although he reported that he identified as heterosexual), and gender identity, which he found distressing. An extensive workup, documented in Table 2, was unremarkable. His urine drug screen 10 with confirmations was positive for marijuana metabolite of 19 ng/mL (>5 ng/mL reference range for positive), to which he reported was due to the mother and patient trying cannabidiol (CBD) oil by mouth to treat his anxiety with reported mild benefit. He remained admitted to the pediatric hospitalist service for a week, during which time provisional diagnoses included psychosis and OCD. He started sertraline daily, olanzapine before bed, and hydroxyzine as needed for anxiety. His symptoms showed minimal improvement.

Due to continued neuropsychiatric symptoms, he was admitted to our inpatient psychiatric unit for 10 days. During the inpatient psychiatric admission, sertraline and olanzapine were further titrated. For further diagnostic clarification, he underwent formal psychological testing, which suggested OCD, major depressive disorder (MDD), generalized anxiety disorder, and autism spectrum disorder (ASD). The instruments used for testing included the Patient Health Questionnaire (PHQ-9), which indicated strong symptoms of a depressive episode in all categories; the Revised Children's Manifest Anxiety Scale (R-CMAS), which strongly suggested generalized anxiety symptoms; the Yale-Brown Obsessive-Compulsive Scale (Y-BOCS), with a total score of 35, indicating severe to disabling OCD; the Social Responsiveness Scale-Second Edition (SRS-2) completed by patient's parents, with moderate ranges for restricted communication and interaction, restricted interest and repetitive behavior; and the Childhood Autism Rating Scale-Second Edition (CARS-2) that indicated mild-to-moderate symptoms of ASD.

During his inpatient psychiatric hospitalization, his symptoms showed moderate improvement, and he was able to create to a safety plan before being discharged home. Following discharge, he established care with outpatient child and adolescent psychiatry. He was recommended to continue olanzapine and hydroxyzine, to start guanfacine for anxiety, impulsivity, and nightmares, and to cross-taper and titrate sertraline to fluvoxamine to target OCD symptoms. He reported improvement in sleep and nightmares but worsening of anxiety and intrusive thoughts. There was concern for catatonia, and clonazepam was trialed, which unfortunately, led to worsening of urges and obsession symptoms and was discontinued.

One year after his first infection with COVID-19 (3 months after his confirmed reinfection), the patient returned to the ED for episodes of aggressive urges and was observed for 15 days. During his observation, he continued fluvoxamine titration with limited improvement. Following discharge, he returned to the ED and was observed for 3 days before he was discharged home. By his next outpatient psychiatry visit, he had reached high doses of fluvoxamine. His school performance deteriorated, and despite several outpatient psychiatry visits, he reported limited improvement in symptoms. He was switched from olanzapine to risperidone, hydroxyzine was titrated, and guanfacine was weaned. He reported fluctuation of symptoms as both risperidone and hydroxyzine were further titrated. Throughout the entire course of his symptoms, he has repeatedly reported his symptoms as severely ego-dystonic. He maintained that the intrusive thoughts and aggressive urges go against “who he is and everything he stands for.” He continues to be seen by the outpatient psychiatry team.

In summary, the patient has a complex constellation of symptoms that, aside for OCD, do not easily support any previously known syndrome or diagnosis. Apart from rigid thinking, dyslexia, possible auditory processing challenges in early childhood, and subtle difficulties with math, his presenting signs and symptoms were brought on and exacerbated in the context of recurring COVID infections. Extensive workups for inflammatory and neurological processes have been inconclusive.

The intrusive thoughts with urges to break things were not behavioral but rather similar to an obsessive–compulsive process, as both the thoughts and urges were ego-dystonic. His thoughts were not disorganized, urges and behaviors were short in duration, and perceptual abnormalities had been brief and fleeting despite antipsychotic therapies or doses, all inconsistent with a specific psychotic disorder. Considering his intrusive thoughts and how pervasive they were, this had likely contributed to his paranoid thinking of “something bad is going to happen” to his family. Therefore, continual monitoring for psychotic processes remained prudent.

At our final appointment (prior to a move out of state), the patient had tolerated risperidone at 3 mg, with stabilization and resolution of tongue tardive and tremor. The decrease of risperidone to 2 mg made urges more difficult to control. His persistent daytime fatigue can still be attributed to the risperidone and divalproex sodium, which worsened at higher doses. There was no benefit with the fluvoxamine at 300 mg, so that was decreased to a beneficial dose to reduce any side effects of sedation at the higher dose. Hydroxyzine, ibuprofen, and turmeric PRN had been calming to the patient, suggesting a potential underlying inflammatory process, and their benefits should continue to be monitored. Lastly, his ongoing anxiety and depression did not represent a specific disorder but rather coincided with his OCD symptoms. Follow-up should continue to assess for prodrome and development of a primary psychotic disorder.

## 3. Discussion

This patient presents a complicated diagnostic picture, and determining the etiology for his symptoms has proven very difficult. Furthermore, he has been largely unresponsive to treatment, further complicating his clinical course. One overarching aspect to his constellation of symptoms is ego-dystonia, thus keeping OCD on his differential. Though the onset of symptoms was acute, as often is consistent with cases of catatonia and/or delirium, his symptoms have steadily worsened and have remained refractory to treatment. Specifically, when clonazepam was administered, the patient's symptoms did not improve, moving catatonia further down on the differential for explaining his symptoms. Additionally, this patient has significant risk factors for severe mental illness, including a family history of schizophrenia, mood disorders, and suicide. His maternal grandfather was diagnosed with schizophrenia and later died by suicide. There is also a paternal history of obsessive–compulsive “tendencies,” consistent with a concern for a family history of mild ASD.

The leading psychiatric differential diagnoses vacillated between OCD and a primary thought disorder/psychosis (see Table 3), with strong support for OCD from the psychological testing. Although the signs of restriction with social communication and interaction could be indicative of prodromal features associated with a psychotic disorder, these could also be seen in an individual with his premorbid history of rigid thinking, dyslexia, and an auditory processing disorder. These symptoms also are consistent with ASD, and the psychological testing identified these mild to moderate symptoms. Alternatively, his hallucinations were largely hypnopompic and transient and did not initially warrant treatment with antipsychotic. He did have reported symptoms of ideas of reference versus delusions, which are seen in a primary thought disorder. Additionally, his persistent avolition and cognitive challenges with schoolwork could also be seen in a primary thought disorder. Although many of the above symptoms can also be seen in MDD or another affective disorder, they were persistent and not episodic, suggesting more of a chronic disorder such as OCD, ASD, or a primary thought disorder. Furthermore, he had little improvement in his mood despite multiple medication trials that should have targeted MDD (or other affective disorder) symptoms.

The distinctive features in this patient associated with the top diagnoses of consideration are presented in Table 3. Of note, while many OCD features were a part of his presenting symptoms, including intense obsessions and compulsions, our patient's emerging symptoms of delusions and derealization, along with his presenting age and family history of schizophrenia, continued to increase the concern for an emerging primary psychotic disorder.

With the acute onset of symptoms following his initial infection with COVID-19, there has been enduring concern for a possible organic etiology for his symptoms secondary to COVID-19. Cases of COVID-19 infections associated with temporary psychosis have been reported in adults and adolescents without significant psychiatric history [4–6]. However, in these cases, the symptoms of psychosis appear to resolve within 90 days, suggesting COVID-19-associated psychosis may be time-limited. Neurological complications secondary to COVID-19 infections, both acute and chronic, are well established and provide evidence to suggest a neuroinflammatory or autoimmune-mediated process associated with the virus [8, 9]. Additionally, our patient's reinfection with COVID-19 a year later, associated with subsequent worsening of symptoms leading to hospitalization, suggests a potential temporal response to the virus. The temporary benefit associated with ibuprofen and hydroxyzine may also suggest an inflammatory mechanism or antibody-mediated encephalitic event. Cases of sudden onset OCD/restrictive eating practices following COVID-19 infections further support the hypothesis that COVID-19 infections can precipitate and/or exaggerate neuropsychiatric sequelae [10–12].

Of notable importance, the patient had been exposed to prednisone and CBD oil during the course of his illness. The patient was initially treated on day 24 from his initial COVID-19 infection with prednisone for his respiratory symptoms. Further complicating his differential diagnosis, prednisone is known to carry its own psychiatric risks, including symptoms of emotional lability, depression, anxiety, and more rarely, hypomanic symptoms and psychotic features. Notably, 7 days after completion of this intervention, the patient's symptoms continued to worsen and did not improve after prednisone was stopped. Most of the reported prednisone-associated psychiatric symptoms appear with chronic use, often at the third week of therapy, and were generally mild and reversible [13], for which this course was not seen in our patient. Regarding the patient's reported CBD oil use for anxiety prior to his first inpatient hospitalization on day 319, he related that this use was brief and was discontinued prior to hospitalization. Although it is possible that he had used additional forms of CBD or Delta-9-tetrahydrocannabionol, we did not think that his reported use and level of exposure was causative of his presenting symptoms given that his stated use was acute, low potency/concentration, and his obsessions preceded this use and continued for months after use, despite reported discontinuation and negative drug screen 103 days later. Nevertheless, it is important to note that acute intoxication with cannabinoids can present with tachycardia, slurred speech, and ataxia, as well as the neuropsychiatric effects of altered mood, perception, thought content, cognition, and psychomotor performance. In many reported cases, these symptoms depended on potency, amount, and route and are often resolved within 24–48 hr [14].

Parental involvement in this case warrants consideration as well. Over the course of 10 months, our patient visited his pediatrician five times and the ED four times with recurrent affective and thought-related complaints, for which he received a standard workup and treatment with a selective serotonin reuptake inhibitor (SSRI). As these complaints continued to worsen, his family grew increasingly concerned, finally driving over an hour to a pediatric-specific ED. It was their persistence and advocacy that helped the patient access a multidisciplinary treatment approach, including assessment by child and adolescent psychiatry, pediatric neurology, and pediatric rheumatology. The subsequent workup described in Table 2, while largely unremarkable, represents a dramatic expansion from the standard workup received at previous visits with a broadened differential. Without his family's ability to advocate for him, it is unlikely this diagnostic pursuit would have occurred. This raises the question: *How many children without this degree of parental involvement*, *may have treatable causes for similar behavior changes that remain undiagnosed?* Additionally, his family's diligent pursuit of a nonpsychiatric diagnosis raises concerns about the introduction of bias, particularly given that his family was responsible for reporting many portions of the history. This introduces the potential for recall bias to link our patient's symptoms with a seemingly more treatable and less stigmatizing diagnosis. Our treatment team may have also introduced bias during the patient's initial hospitalization through the phenomenon of positive countertransference. This patient and his family were very well-liked by our treatment team. We have considered whether our team was initially driven to avoid diagnoses with a poorer prognosis, such as psychosis/schizophrenia, in favor of potentially more hopeful and less stigmatizing diagnoses, such as OCD, pediatric acute-onset neuropsychiatric syndrome, or long-COVID.

There are significant limitations to this report. A single case report does not represent enough data to establish a trend and is also insufficient to establish causality. Treatment modalities reported here are limited to antipsychotics, SSRIs, mood stabilizers, benzodiazepines, and anticholinergic agents. Other possible treatment modalities, including prolonged, high-dose steroids, and intravenous immunoglobulin, were not pursued due to the lack of evidence of inflammation or autoimmune illness to support their use. An alternative limitation is that while the Mayo Autoimmune Encephalitis panel represents the gold standard for evaluation of autoantibodies known to cause neuropsychiatric complications, there may be other undiscovered, and therefore untested, autoantibodies mediating his illness.

This case provides insight into the challenges of diagnosing and treating a disparate constellation of symptoms complicated by a temporal association with a novel virus. It is important for researchers to continue to investigate neuropsychiatric complications associated with COVID-19 to better understand this phenomenon and work toward improving care.

## Figures and Tables

**Table 1:** Time of medical encounters and progression of symptoms.

Location	Treatment Day	Symptoms	Labs/Images	Treatment
Urgent care	1	Shortness of breath and cough	COVID-19 PCR	
Outpatient pediatrics	24	Shortness of breath, nasal congestion, and anxiety		Prednisone 60 mg × 5 days
Outpatient pediatrics	37	Worsening anxiety, hopelessness, inattention, poor sleep, and tearfulness		Escitalopram 10 mg daily
Outpatient pediatrics	50	Worsening anxiety, distorted thoughts, fear of being alone, fear of taking showers, racing thoughts, confusion, intrusive thoughts	CBC, CMP, TSH, CRP	Referral to counseling
Emergency department	152	Worsening depressive thoughts, suicidal ideation, and thoughts of hurting others	CBC, CMP, ethanol, UDS	
Outpatient pediatrics	177	Daily headaches, “brain fog,” difficult sleep onset, waking up during night, auditory hallucinations, tearfulness	CBC, CMP, TSH, LDH, uric acid, troponin, CT head	
Outpatient pediatrics	233	Difficulty with thought organization, “brain fog,” derealization		
Emergency department	285	Punching the ground with his fist, confusion, rapid speech, visual hallucinations	CBC, CMP, ethanol, UDS	
Emergency department	292–297	Rage, punching walls and doors, rapid speech, auditory hallucination, sadness, and hopelessness, feeling “messed up,” lack of control of thoughts, transient SI	CBC, CMP, ethanol, UDS, COVID-19	Tele-psych inpatient observation for 5 days
Outpatient pediatrics	298	Sore throat, nasal congestion, and shortness of breath	COVID-19 PCR	
Pediatric emergency department	319	Increasingly severe intrusive thoughts, homicidal ideation, “brain fog,” visual hallucinations, delusions, inability to control his thoughts	CBC, CMP, CRP, UDS, Marijuana metabolites	Admitted to inpatient pediatric hospitalist service
Pediatric inpatient hospitalization	319–326	Marked anger, aggressive behavior, obsessions, intrusive homicidal and homosexual thoughts, nonspecific auditory and visual perceptual anomalies, depersonalization, depression, anxiety, and sleep onset difficulty	See Table 3	Sertraline 25 mg daily, Olanzapine 5 mg daily, Hydroxyzine 25 mg PRN
Inpatient psychiatric hospitalization	326–336	Moderate improvement of symptoms	Psychological testing: PHQ-9, R-CMAS, Y-BOCS, SRS-2, CARS-2	Sertraline 200 mg daily, Olanzapine 10 mg daily, Hydroxyzine 25 mg PRN
Emergency department	351–365	Rage, aggressive behavior, intrusive homicidal and homosexual thoughts, passive SI, apathy, anhedonia, depression	CBC, CMP, ethanol, UDS, U/A	Tele-psych inpatient observation for 15 days
Emergency department	391–394	Rage, anxiousness, hopelessness, flattened affect, auditory and visual hallucinations, cognitive slowing	CBC, CMP, ethanol, UDS	Tele-psych inpatient observation for 3 days
Outpatient child and adolescent psychiatry	Days 395–605 includes 13 follow-up appointments	Moderate improvement of symptoms -Psychotic features improved (no AVH, ideas of reference, delusions, or disorganized thoughts) -Has ongoing anxiety and worries that “something bad will happen,” in the context of not being able to defend family -Intrusive thoughts are constant, and urges wax and wane with decreases in divalproex sodium and risperidone -Thoughts are primarily homicidal, which are completely ego-dystonic -Higher doses of risperidone and valproic acid were too sedating -Persistent cognitive difficulties with math and attention	CMP and liver enzymes normalized. Lipid panel, hemoglobin A1C WNL. AIMS-0 had increased tardive/tremor of the tongue at risperidone 5 mg and improved at 3 mg.	Fluvoxamine 250 mg QHS, divalproex sodium ER 500 mg QHS, Risperidone 3 mg QHS Hydroxyzine 75 mg QHS for anxiety and sleep. Mother gives turmeric OTC and ibuprofen 400 mg OTC when urges worsen, with a notable “calming” effect

**Table 2:** Laboratory and imaging evaluation.

Differential diagnosis	Test(s)	Treatment day	Result
Autoimmune encephalitis	CSF^c^ cell count with differential	322	Red blood cells 133 cells/*μ*L (traumatic tap)
	Mayo autoimmune encephalitis panel ENC2	323	Negative
	MRI^d^	319	Noted below^e^
		421	Stable from previous
*Bartonella henselae*	*B. henselae* antibodies	324	Negative
Hepatic encephalopathy	Ammonia NH_3_	439	Negative
	Hepatitis panel		Negative
	Amino acids		Negative
Hyperlipidemia	Lipid panel	421	TG^i^ 169 mg/dL (high) HDL^j^ 36 mg/dL (low) LDL^k^ 71 mg/dL (normal)
Hyperthyroidism	Thyroid-stimulating hormone	50	1.35 *μ*IU/mL
		319	2.29 *μ*IU/mL (normal)
	Thyroid peroxidase autoabs^h^	439	Negative
	Thyroglobulin autoabs		Negative
Lyme disease	Lyme disease antibodies	324	Negative
Marijuana use disorder	Urine drug screen	319	Positive cannabinoids
	Marijuana metabolites qnt		19 ng/mL (>5 ng/mL limit)
Meningitis	CSF meningoencephalitis PCR^g^	322	Negative
Multiple sclerosis	CSF oligoclonal bands	322	Negative
PANDAS^b^	Beta strep group A culture	316	Negative
SARS-CoV-2 infection sequelae	SARS-CoV-2 antibodies	439	Positive
	SARS-CoV-2 IgG II qnt^a^		1,537.8 AU/mL
	T and B-cell enumeration panel		CD3, CD4 (low) CD8,19,16,56 (normal)
	SARS-CoV-2 antigen	298	Positive
Substance use disorder/intoxication	Urine drug screen	177	Negative
		421	Negative
		479	Negative
Systemic lupus erythematosus	ANA IFA^f^ with reflex to titer	386	Negative
Wilson's disease	Ceruloplasmin	421	Negative

^a^SARS-CoV-2 IgG II quantitative antibody assay. ^b^Pediatric autoimmune neuropsychiatric disorder associated with group A streptococci. ^c^Cerebrospinal fluid. ^d^Magnetic resonance imaging. ^e^“Multiple small foci of T2/FLAIR hyperintense signal in the left greater than right frontal subcortical white matter, left frontal corona radiata, and left frontal periventricular white matter. Overall, these lesions, especially in the subcortical white matter, are of uncertain clinical significance and may reflect gliosis from a variety of nonspecific remote insults. Appearance of the lesions in the left frontal periventricular white matter, however, also raises the possibility of nodular periventricular gray matter heterotopia”. ^f^Antinuclear antibodies using immunofluorescence assay. ^g^Cerebrospinal fluid meningoencephalitis polymerase chain reaction. ^h^Thyroid peroxidase autoantibodies. ^i^Triglycerides. ^j^High-density lipoprotein. ^k^Low-density lipoprotein.

**Table 3:** Distinct patient symptoms and differential diagnoses.

Differential diagnosis	Patient symptoms
Obsessive–compulsive disorder	Ego-dystonic intrusive thoughts (with HI ^*∗*^) and urges (compulsions) to act out to control intrusive thoughts (not repetitive or ritualistic)
	Obsession of recurrent, unwanted, intrusive thoughts that were violent in nature, to self, objects, and others, sometimes sexual (of self), and sometimes religious
	Difficult to control compulsions and urges to act aggressively, self-injury, isolate
	Moderate improvement with fluvoxamine, worsened with clonazepam

Psychosis	Decreased socialization (prodromal)
	Level of functioning impacted—school, martial arts, interpersonal relations, and failure to achieve expected level of performance
	Flat affect
	Delusions
	Ideas of reference
	Sensing the presence of an unseen person, “someone reaching out to me”
	Anosognosia
	Family history of schizophrenia
	Age of presentation
	Moderate improvement with antipsychotic

Affective disorder	Racing thoughts
	Flat affect
	Hopeless
	Sadness
	Intermittent suicidal ideation
	Moderate improvement with SSRI

Catatonia	Flat affect
	Decreased movement
	Decreased engagement with the external environment
	Acute onset
	Symptoms did not improve with clonazepam (inconsistent with catatonia)

## Data Availability

The authors confirm that the data supporting the findings of this case report are available within the article and its supplementary materials.
